# LexR Positively Regulates the LexABC Efflux Pump Involved in Self-Resistance to the Antimicrobial Di-*N*-Oxide Phenazine in Lysobacter antibioticus

**DOI:** 10.1128/spectrum.04872-22

**Published:** 2023-05-11

**Authors:** Yangyang Zhao, Gaoge Xu, Zhizhou Xu, Baodian Guo, Fengquan Liu

**Affiliations:** a Institute of Plant Protection, Jiangsu Academy of Agricultural Sciences, Jiangsu Key Laboratory for Food Quality and Safety, State Key Laboratory Cultivation Base of Ministry of Science and Technology, Nanjing, China; b School of Plant Protection, Key Laboratory of Green Prevention and Control of Tropical Plant Diseases and Pests, Ministry of Education, Hainan University, Haikou, China; c College of Plant Protection, Key Laboratory of Integrated Management of Crop Diseases and Pests, Nanjing Agricultural University, Nanjing, China; University of Minnesota Twin Cities

**Keywords:** myxin, phenazine, *Lysobacter antibioticus*, self-resistance, efflux pump, LysR family regulator

## Abstract

Myxin, a di-*N*-oxide phenazine isolated from the soil bacterium Lysobacter antibioticus, exhibits potent activity against various microorganisms and has the potential to be developed as an agrochemical. Antibiotic-producing microorganisms have developed self-resistance mechanisms to protect themselves from autotoxicity. Antibiotic efflux is vital for such protection. Recently, we identified a resistance-nodulation-division (RND) efflux pump, LexABC, involved in self-resistance against myxin in *L. antibioticus*. Expression of its genes, *lexABC*, was induced by myxin and was positively regulated by the LysR family transcriptional regulator LexR. The molecular mechanisms, however, have not been clear. Here, LexR was found to bind to the *lexABC* promoter region to directly regulate expression. Moreover, myxin enhanced this binding. Molecular docking and surface plasmon resonance analysis showed that myxin bound LexR with valine and lysine residues at positions 146 (V146) and 195 (K195), respectively. Furthermore, mutation of K195 *in vivo* led to downregulation of the gene *lexA*. These results indicated that LexR sensed and bound with myxin, thereby directly activating the expression of the LexABC efflux pump and increasing *L. antibioticus* resistance against myxin.

**IMPORTANCE** Antibiotic-producing bacteria exhibit various sophisticated mechanisms for self-protection against their own secondary metabolites. RND efflux pumps that eliminate antibiotics from cells are ubiquitous in Gram-negative bacteria. Myxin is a heterocyclic *N*-oxide phenazine with potent antimicrobial and antitumor activities produced by the soil bacterium *L. antibioticus*. The RND pump LexABC contributes to the self-resistance of *L. antibioticus* against myxin. Herein, we report a mechanism involving the LysR family regulator LexR that binds to myxin and directly activates the LexABC pump. Further study on self-resistance mechanisms could help the investigation of strategies to deal with increasing bacterial antibiotic resistance and enable the discovery of novel natural products with resistance genes as selective markers.

## INTRODUCTION

Phenazines are heterocyclic *N*-containing redox natural products with a wide range of biological activities, including antimicrobial and antitumor activities (1–3). Further, phenazines play an important role in the persistence and survival of their producers in the rhizosphere (4–6). The first identified phenazine, pyocyanin, was followed by the identification of >180 natural phenazines, mainly produced by Pseudomonas and *Streptomyces* (1, 3). A widely known and studied phenazine, phenazine-1-carboxylic acid (PCA), from Pseudomonas spp. was registered as a biopesticide in China in 2011. The compound is commercially called Shenqinmycin and effectively prevents and controls various fungal, bacterial, and nematode diseases (7, 8). Other active natural phenazines could also be developed as biopesticides.

*Lysobacter* is a Gram-negative bacterial genus that includes species that produce many active extracellular enzymes and secondary metabolites. Members of this genus have recently attracted considerable attention as sources of biocontrol agents (9–11). Our previous study isolated six phenazines from Lysobacter antibioticus OH13, and the phenazine *N*-oxide myxin exhibited strong antimicrobial activity (12, 13). Myxin is a heterocyclic aromatic *N*-oxide, a chemical class rarely found in the environment, and can cause DNA damage when bioreductively activated (14, 15).

High concentrations of myxin are toxic to *L. antibioticus* OH13, the strain that produces the chemical. Thus, self-toxicity may limit the production of myxin. Antibiotic-producing microorganisms exhibit multiple resistance mechanisms to prevent self-toxicity effects, such as antibiotic efflux, inactivation, and target repair or protection (16). Recently, we identified a resistance-nodulation-cell division (RND) efflux pump, LexABC (17), and a monooxygenase, LaPhzX (18), involved in self-resistance to myxin in *L. antibioticus* OH13.

RND efflux pumps in Gram-negative bacteria exhibit a wide spectrum of substrates and have an important role in bacterial multidrug resistance. These efflux pumps consist of three proteins: an inner membrane RND transporter, outer membrane protein, and plasma membrane fusion protein (19, 20). Twelve RND efflux pumps are recognized in Pseudomonas aeruginosa; 11 are capable of multidrug efflux, including MexAB-OprM, MexCD-OprJ, MexEF-OprN, MexXY, and MexGHI-OpmD. These pumps are responsible for β-lactam, aminoglycoside, fluoroquinolone, and phenazine efflux (19, 21). RND efflux pumps are often regulated by two-component systems and individual TetR, LysR, MarR, and AraC family proteins (22). LysR-type transcriptional regulators are abundant in bacteria. The MexEF-OprN RND efflux pump from P. aeruginosa can be activated by MexT, a transcriptional regulator of this family. Activation occurs when cells encounter electrophilic substances, and regulation depends on the presence of a putative quinol monooxygenase (PA2048) and quinone oxidoreductase (MexS) (23, 24). AmpR, another member of the LysR family, regulates non-β-lactam antibiotic resistance by modulating the MexEF-OprN efflux pump (25). Yet another LysR-type transcriptional regulator, AdeL, negatively regulates the AdeFGH RND efflux system (26). LysR regulators involved in RND efflux pump expression are ubiquitous, but their molecular mechanisms and responses to antibiotics are not clear.

In *L. antibioticus* OH13, deletion of *lexABC* genes greatly increases the susceptibility of strains to myxin and decreases myxin production, and the expression of *lexABC* is induced by myxin. A putative LysR family protein-encoding gene, *lexR*, located upstream of *lexABC*, decreases myxin resistance and production when mutated. A deletion mutant of *lexR* causes downregulation of *lexABC* (17). LexR positively regulates the LexABC pump, although its action on a molecular scale has not been determined. We elucidated its mechanism of action, and we explain mechanisms behind the myxin upregulation of *lexABC* expression. These findings confirm the regulation of myxin efflux and provide insight into the self-resistance mechanism against myxin.

## RESULTS

### Determination of the *lexQSABC* operon.

**FIG 1:**
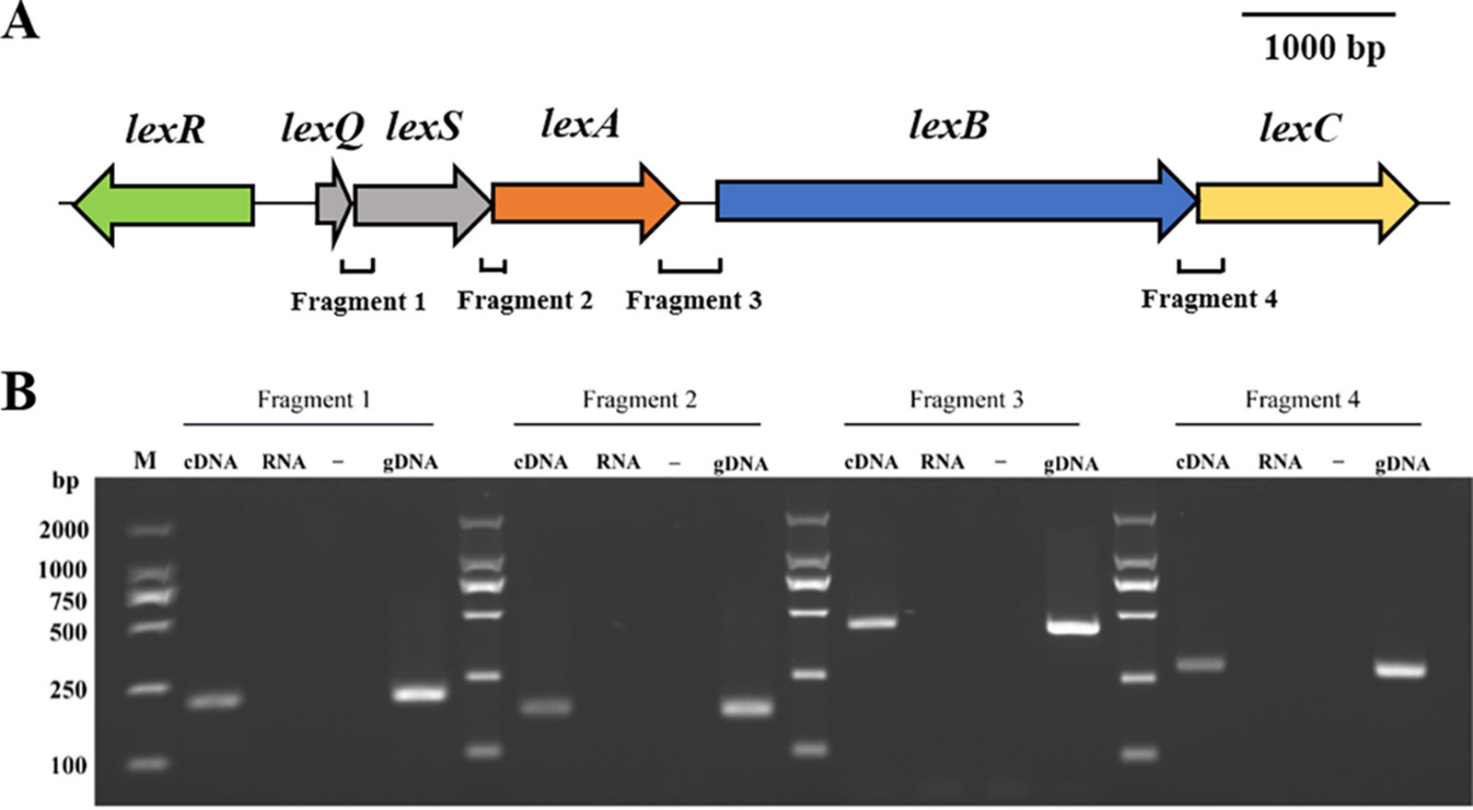
Determination of the *lexQSABC* operon. (A) Linear map of *lexQ*, *lexS*, *lexA*, *lexB*, and *lexC* genes with the genomic DNA of *L. antibioticus* OH13. (B) Results of RT-PCR assays to verify the coexpression of *lexQ* to *lexC*. cDNA, gDNA, and RNA were used as templates for PCR amplification. −, negative control; M, DNA marker. Fragment 1, 204 bp; fragment 2, 168 bp; fragment 3, 408 bp; fragment 4, 280 bp.

The organization of the *lexABC* cluster and the gene *lexR* were described in our previous study (17). There are two other genes located closely upstream of *lexABC*; here, we have named them *lexQ* and *lexS*, respectively. The *lexS* gene overlaps *lexA* by 4 bp and is 8 bp immediately downstream of *lexQ* (Fig. 1A). Reverse transcription PCR (RT-PCR) was performed with complementary DNA (cDNA) synthesized using RNA from *L. antibioticus* OH13 to assess the coexpression of *lexQ*, *lexS*, *lexA*, *lexB*, and *lexC* genes. Fragments 1, 2, 3, and 4 from the two adjacent genes were amplified from cDNA and genomic DNA (gDNA) but not from RNA or the negative control (Fig. 1B). Thus, the genes from *lexQ* to *lexA* formed an operon, designated *lexQSABC*.

### LexR directly activates *lexABC* expression.

**FIG 2:**
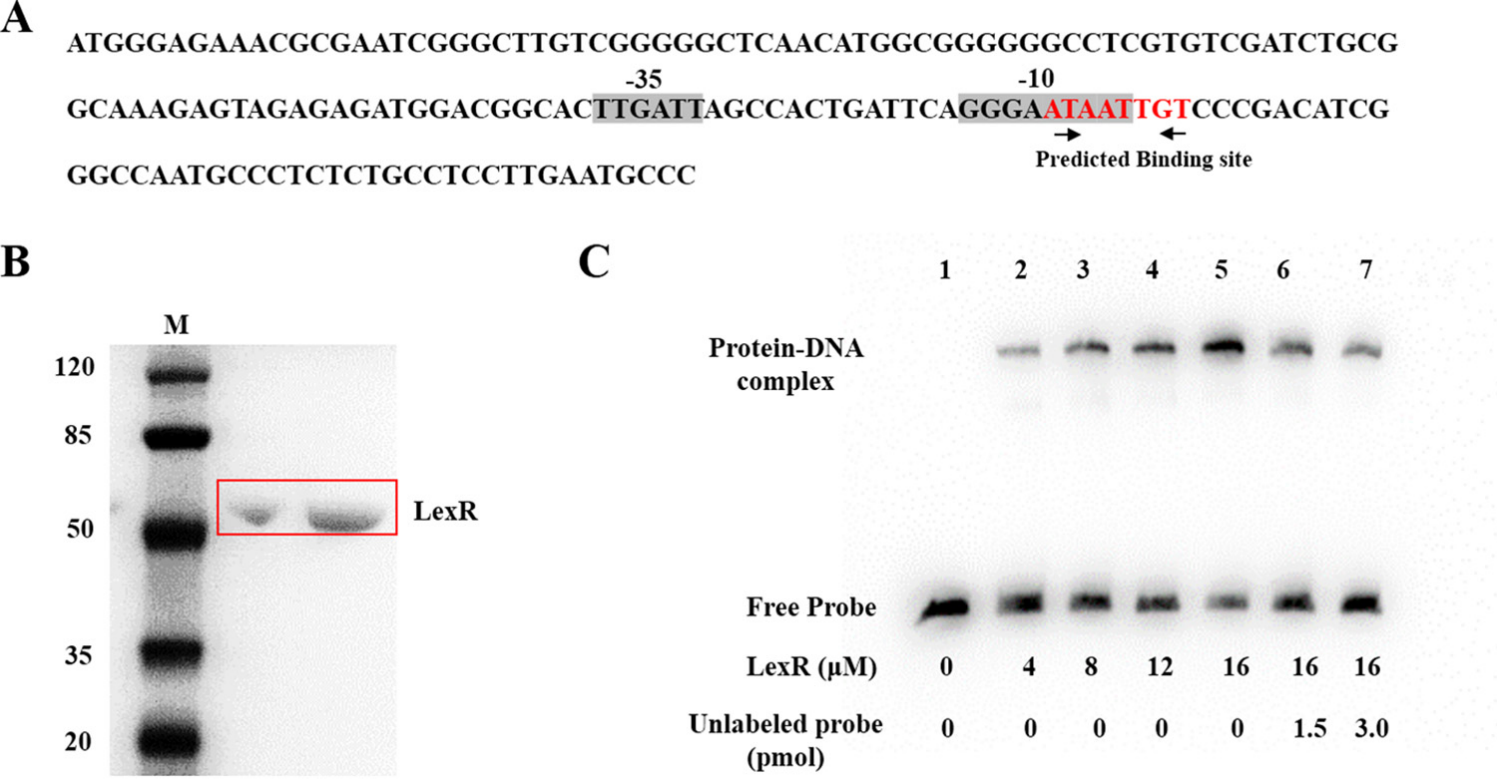
LexR directly regulates LexABC expression. (A) The predicted promoter region of the *lexQSABC* operon. The putative −35 and −10 sequences are shaded. The putative binding site is shown in red. (B) SDS-PAGE results for the His-tagged, purified LexR (53.1 kDa). (C) EMSA results, showing binding of LexR to the *lexQSABC* promoter region *in vitro*. A 165-bp biotin-labeled probe (*lex* probe) was incubated with increasing amounts of LexR protein (concentrations are indicated). Lane1, 5 fmol labeled probe; lanes 2 to 5, 5 fmol labeled probe with different concentrations of LexR; lanes 6 and 7, 5 fmol labeled probe and different concentrations of unlabeled probe with LexR.

Deletion of *lexR* increased sensitivity to myxin, decreased myxin production, and significantly downregulated *lexABC* (17). Hence, we hypothesized that LexR directly regulates these genes. A putative promoter region found by the online promoter prediction tools BPROM may drive this coexpression (Fig. 2A; see also the supplemental material). An electrophoretic mobility shift assay (EMSA) was used to determine if LexR directly activates *lexABC* expression by binding to the promoter upstream of *lexQ*. A 165-bp DNA probe (*lex* probe) was amplified with primers labeled with biotin at the 5′ end. LexR with a His tag was expressed in Escherichia coli BL21(DE3) and purified using affinity chromatography. SDS-PAGE showed a single protein band with a molecular mass of ~53.1 kDa, indicating that LexR was successfully expressed and purified for subsequent EMSA (Fig. 2B). EMSA results indicated that purified LexR bound to the biotin-labeled *lex* probe, which reduced its mobility (Fig. 2C). Moreover, DNA-protein-binding bands were enhanced with increasing concentrations of LexR. This phenomenon was greatly inhibited by unlabeled promoter (Fig. 2C). We used a 158-bp promoter region (control probe) from another RND efflux pump operon to perform the control experiment. The result showed that LexR did not bind a probe of similar length from an unrelated promoter region (Fig. S1). Thus, the regulator LexR specifically binds to the promoter region of the *lexQSABC* operon.

**FIG 3:**
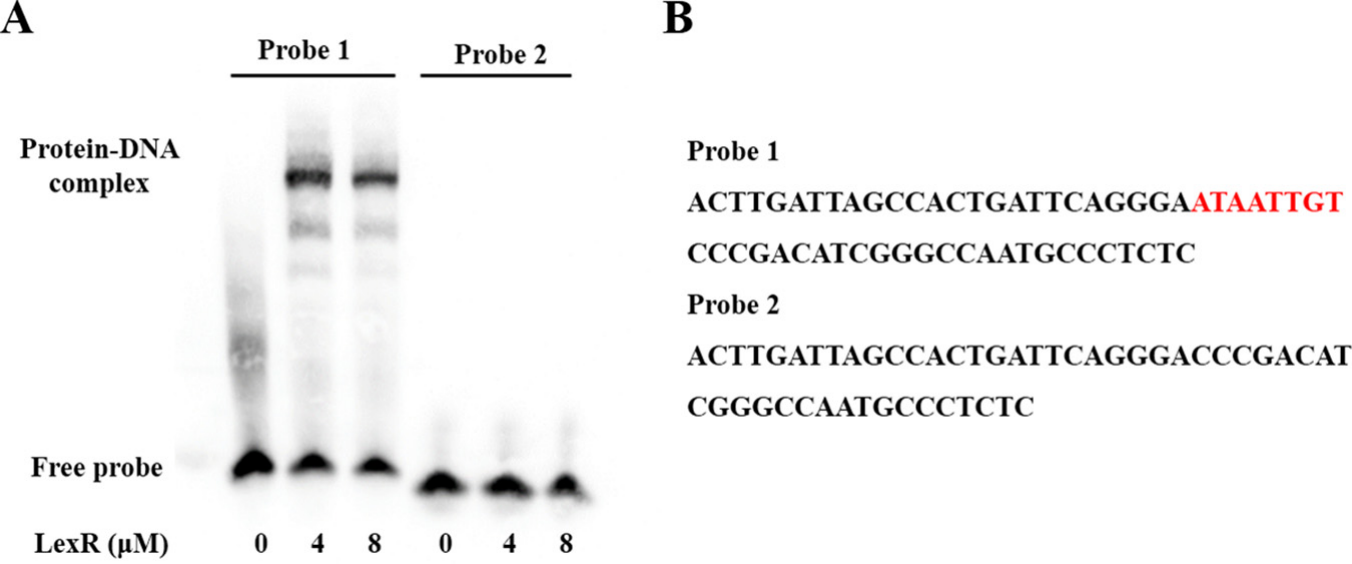
Confirmation of the necessary sequence in the promoter region of *lexQSABC* for LexR binding. (A) EMSA results, indicating an 8-bp sequence necessary in the *lexQSABC* promoter for LexR binding. Five femtomole of labeled probe 1 and probe 2 were used, and the concentrations of LexR are indicated. Probe 1, a 58-bp biotin-labeled probe from the *lexQSABC* promoter region; probe 2, a 50-bp biotin-labeled probe with a sequence 8 bp less than probe 1 sequence. (B) Sequences of probes 1 and 2. The red bases are those necessary for LexR binding.

The online prediction revealed a putative LexR binding site in the *lexQSABC* promoter region (Fig. 2A). Thus, we used 58-bp (probe 1) and 50-bp (probe 2) biotin-labeled probes with and without this site, respectively, to examine the need of this site for LexR binding. Subsequently, EMSA was carried out with purified LexR and probes 1 and 2, which showed that LexR bound to probe 1 but not to probe 2 (Fig. 3). To further confirm that the predicted binding sequence for LexR is necessary or sufficient, we performed another experiment. Probe 3 (58 bp from the control probe in Fig. S1) and probe 4 (which replaced the predicted binding sequence to probe 3) were synthesized and then incubated with LexR in an EMSA (Fig. S2). The result showed that addition of the putative binding sequence to probe 3 could not sufficiently induce its binding to LexR (Fig. S2). Thus, the 8-bp putative binding sequence is necessary but not sufficient for LexR binding to the promoter region.

### Myxin enhances LexR binding to the *lexABC* promoter.

**FIG 4:**
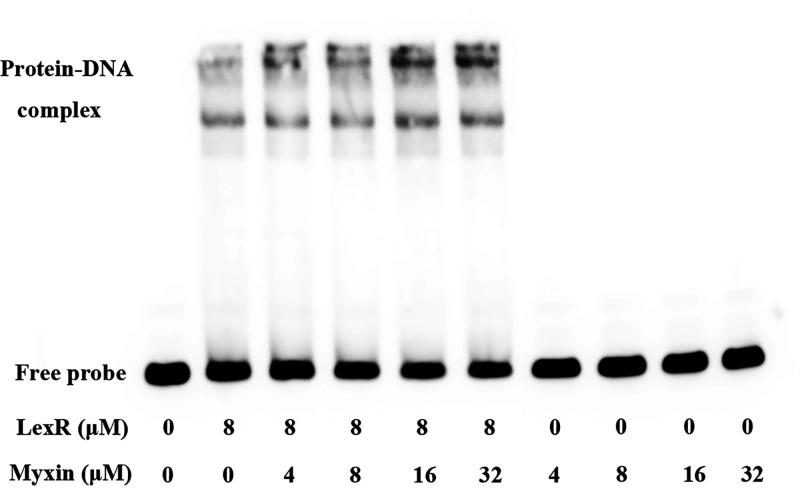
Myxin enhanced the binding of LexR to the *lexQSABC* promoter region. The reactions were performed with 5 fmol *lex* probe. The concentrations of LexR and myxin are shown. Myxin was incubated with DNA probe as a control.

We determined that myxin could efficiently increase the expression of *lexABC* genes in our previous study (17). So, we next examined whether *lexABC* transcriptional activation by myxin via enhancing LexR-DNA binding. An EMSA in the presence of myxin, LexR protein, and *lex* probe was performed. The result showed increased intensity in the LexR-DNA binding complex with myxin compared to LexR and probe alone but not affecting LexR-DNA migration in the gel. Increasing the myxin concentration resulted in more LexR-DNA complex formation, indicating that myxin enhanced LexR-DNA binding in a concentration-dependent manner (Fig. 4). Control assays were conducted with various concentrations of myxin added to the probes without LexR, which suggested that myxin did not damage the DNA probe under this condition (Fig. 4).

### LexR displays a binding site for myxin.

**FIG 5:**
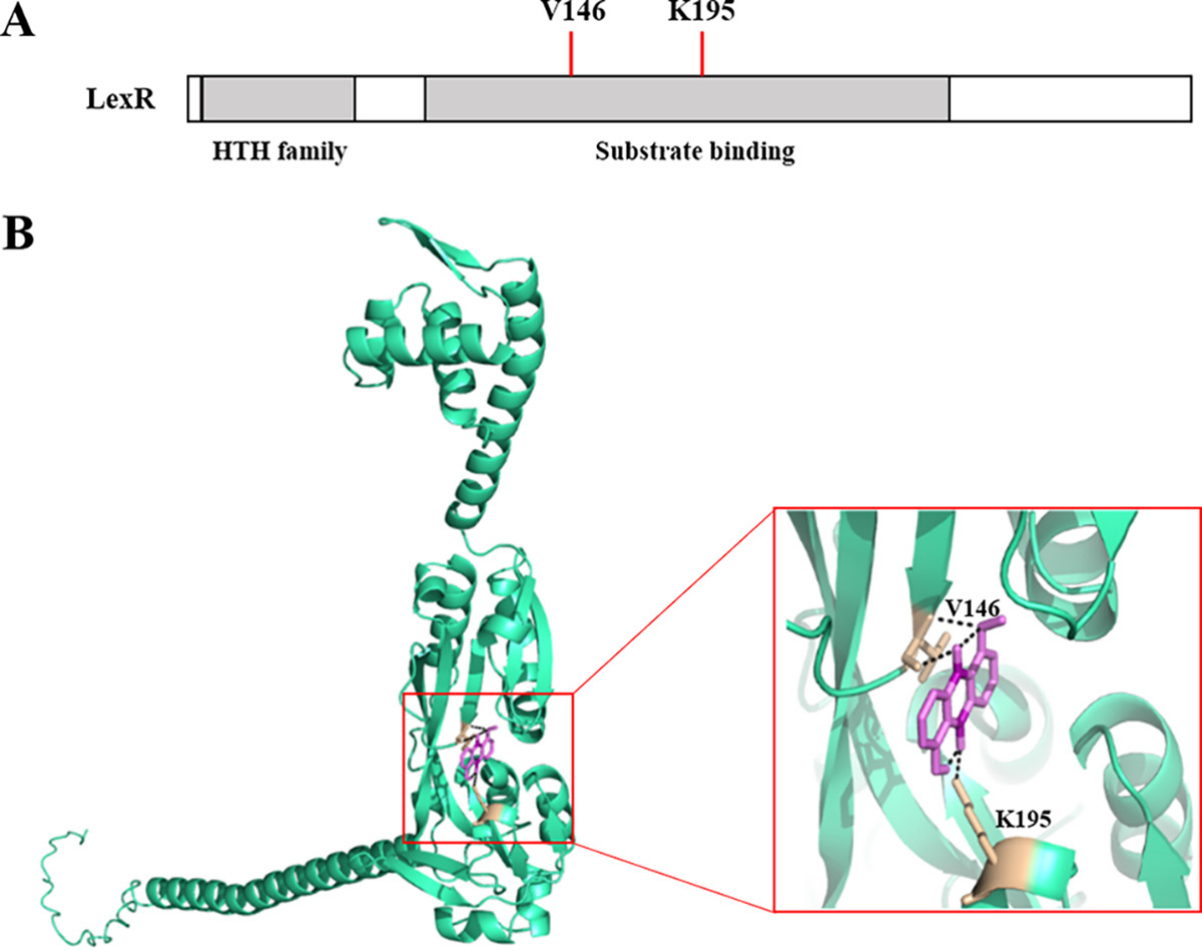
Predicted binding sites of LexR to myxin. (A) Domain structure of LexR. (B) Molecular docking, showing a binding pocket in LexR with myxin. The residues in contact with the ligand are shown as sticks in the model and are labeled.

**FIG 6:**
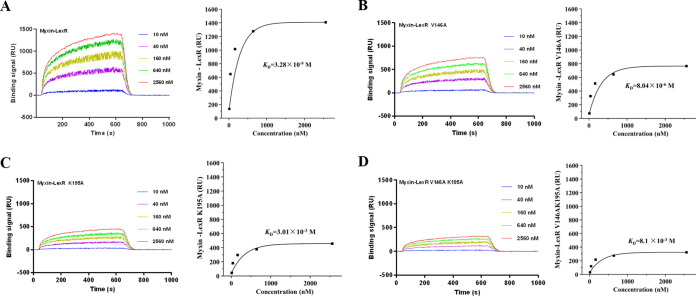
LexR binds to myxin with a binding site. SPR analysis of the affinity fit for myxin binding to LexR (A), LexR-V146A (B), LexR-K195A (C), and LexR-V146A-K195A (D). SPR sensorgrams of myxin binding to different concentrations of proteins are shown on the left, and the fits to the data are presented on the right. The concentrations of proteins and *K_D_* values are indicated.

**FIG 7:**
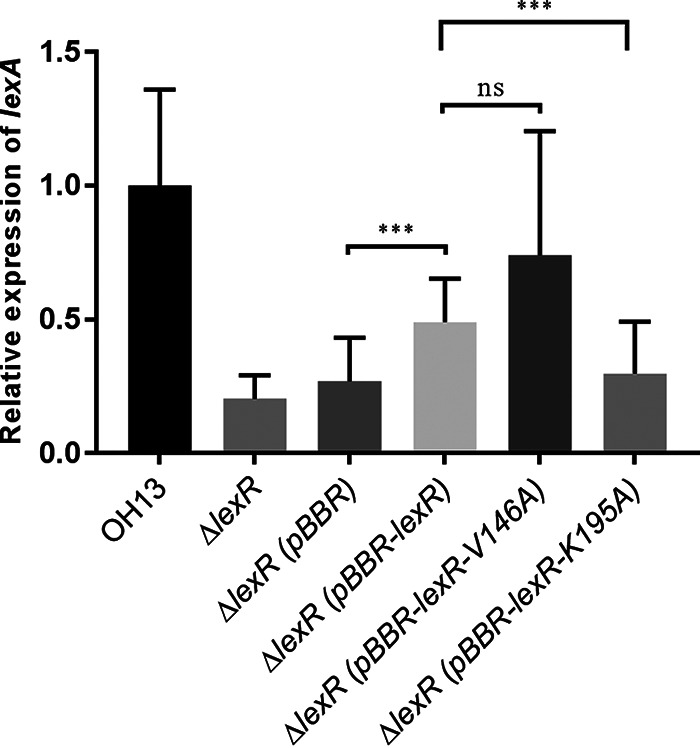
qRT-PCR results, demonstrating that the LexR-myxin complex contributes to *lexABC* transcription. OH13, wild-type strain; Δ*lexR*, *lexR* deletion mutant; Δ*lexR*(*pBBR*), Δ*lexR* carrying the empty pBBR1-MCS5 plasmid; Δ*lexR*(*pBBR*-*lexR*), Δ*lexR* carrying pBBR1-MCS5 with the *lexR* gene; Δ*lexR*(pBBR-*lexR*-V146A), Δ*lexR* carrying pBBR1-MCS5 with V146 mutation of *lexR* gene; Δ*lexR* (pBBR-*lexR-*V195A), Δ*lexR* carrying pBBR1-MCS5 with the K195 mutation of the *lexR* gene. Data are means from three independent experiments, and error bars are standard deviations. Asterisks indicate statistically significant differences according to the *t* test in GraphPad Prism 7.0. ***, *P* < 0.001; ns, not significant.

The three-dimensional structure of LexR predicted by AlphaFold was downloaded from UniProt. Domain prediction and molecular docking showed that the valine residue at position 146 (V146) and lysine at 195 (K195) were likely myxin binding sites (Fig. 5A and B). This docking agrees with surface plasmon resonance (SPR) analysis. Myxin was immobilized on the chip surface, and different LexR concentrations were run over the chip surface. LexR bound myxin with a dissociation constant (*K_D_*) of 3.28 × 10^−9^ M, indicating efficient binding (Fig. 6A). Subsequently, we obtained residue mutants LexR-V146A, LexR-K195A, and LexR-V146A-K195A, to which myxin bound weakly, with *K_D_* values of 8.04 × 10^−6^, 3.01 × 10^−3^, and 8.1 × 10^−3^ M, respectively (Fig. 6B to D). Amino acid residues V146 and K195 are therefore essential for LexR binding by myxin, and K195 was more important for strong interaction. To further quantify the different effect of LexR and LexR-myxin complex on *lexABC* transcription, we constructed LexR site-directed mutants of V146A and K195A *in vivo* and detected the expression level of *lexA* in the mutants via quantitative RT-PCR (qRT-PCR). Gene complementation of *lexR* partially restored *lexA* transcription compared to the *lexR* mutant, and V146A mutant acted identically to the complemented strain, whereas the K195A mutant showed a lower *lexA* expression level than the *lexR* complemented strain (Fig. 7). The results indicated that the LexR-myxin complex contributes to *lexABC* transcription.

## DISCUSSION

Antibiotic-producing microorganisms exhibit effective strategies to avoid self-harm. Studies on self-resistance genes against antibiotics have enabled the discovery of novel natural products with resistance genes as selective markers. These genes have enabled the investigation of strategies to deal with increasing bacterial antibiotic resistance (27–29). Antibiotic efflux via molecular pumps are an effective resistance mechanism. Complex regulation mechanisms are involved in processes that cause the upregulation of efflux pumps (30). Substrates for efflux pumps can frequently influence the expression of pump regulators (31).

Our research demonstrated regulatory links between the regulator LexR, efflux pump LexABC, and myxin in *L. antibioticus*. Mutant *lexR* decreased myxin resistance, myxin production, and *lexABC* expression. Moreover, the expression of *lexABC* increased with the accumulation of myxin *in vivo*, and exogenous addition of myxin to a phenazine-deficient mutant considerably enhanced *lexABC* expression (17). LexR directly and positively regulated the expression of *lexABC* by binding its promoter, and myxin strengthened the binding with LexR as the receptor.

Secondary metabolites, including antibiotics, can act as signaling molecules for the control of gene expression (32). The expression of efflux pumps is often induced by transcription factors that respond to small-molecule inducers (21, 33).

A model regulation system, phenazine/SoxR/MexGHI-OpmD, for natural product efflux and self-protection in antibiotic-producing bacteria has been established in P. aeruginosa. MexGHI-OpmD is upregulated by pyocyanin and its endogenous intermediate 5-methylphenazine-1-carboxylate (5-Me-PCA). The induction of *mexGHI*-*opmD* by phenazine is mediated by activating the redox-active transcription factor SoxR via oxidation or nitrosylation of its [2Fe-2S] cluster (34–36). PCA signals activate *mexGHI*-*opmD* in P. aeruginosa M18, and SoxR mediates the downstream regulation of PCA. A conserved Sox-dependent transcriptional regulatory role likely exists for phenazine pigment efflux (37).

Myxin is an *N*-oxidation and *O*-methylation phenazine, which distinguishes it from pyocyanin, 5-Me-PCA, and PCA. The LexABC pump is similar to MexHI-OpmD, but efflux regulation can differ between and even within bacterial species, depending on cellular physiological status. Activation of *lexABC* is mediated by the LysR family, not a SoxR-type regulator. We propose a specific pathway for the myxin response and efflux in *L. antibioticus*.

Regulators of efflux pumps usually possess a drug-binding pocket within the ligand-binding domain. Binding of drugs or natural products to these regulators modulates their transcriptional activity (38). AdeL, a LysR-type transcriptional regulator, contains a helix-turn-helix domain and substrate-binding domain responsible for negative regulation of the RND efflux pump, AdeFGH, in Acinetobacter baumannii (26, 39). The human pathogen A. baumannii exhibited increased resistance with mutations in *adeL* that induce upregulation of the AdeFGH efflux pump (40). A valine-to-glycine substitution at position 139 of AdeL (V139G) in the signal recognition domain induced overexpression of AdeFGH in A. baumannii mutant strain BM4664 (26). LexR shares 55% similarity with AdeL (17). However, positive regulators of LexABC show a conserved valine at position 146 (26, 41). A valine-to-alanine substitution at this position (V146A) led to weak binding of myxin, further confirming that the conserved valine is a signal recognition site. Another binding residue, K195, is not conserved in LysR family regulators and could be specific for myxin. Moreover, the SPR binding curve and corresponding raw data showed no signal plateau, which indicated a stoichiometry of 1:1 for the myxin-LexR interaction. The *in vivo* site mutation of K195 resulted in less *lexA* transcription which, consistent with the SPR data, supported the importance of the K195 residue for LexR.

**FIG 8:**
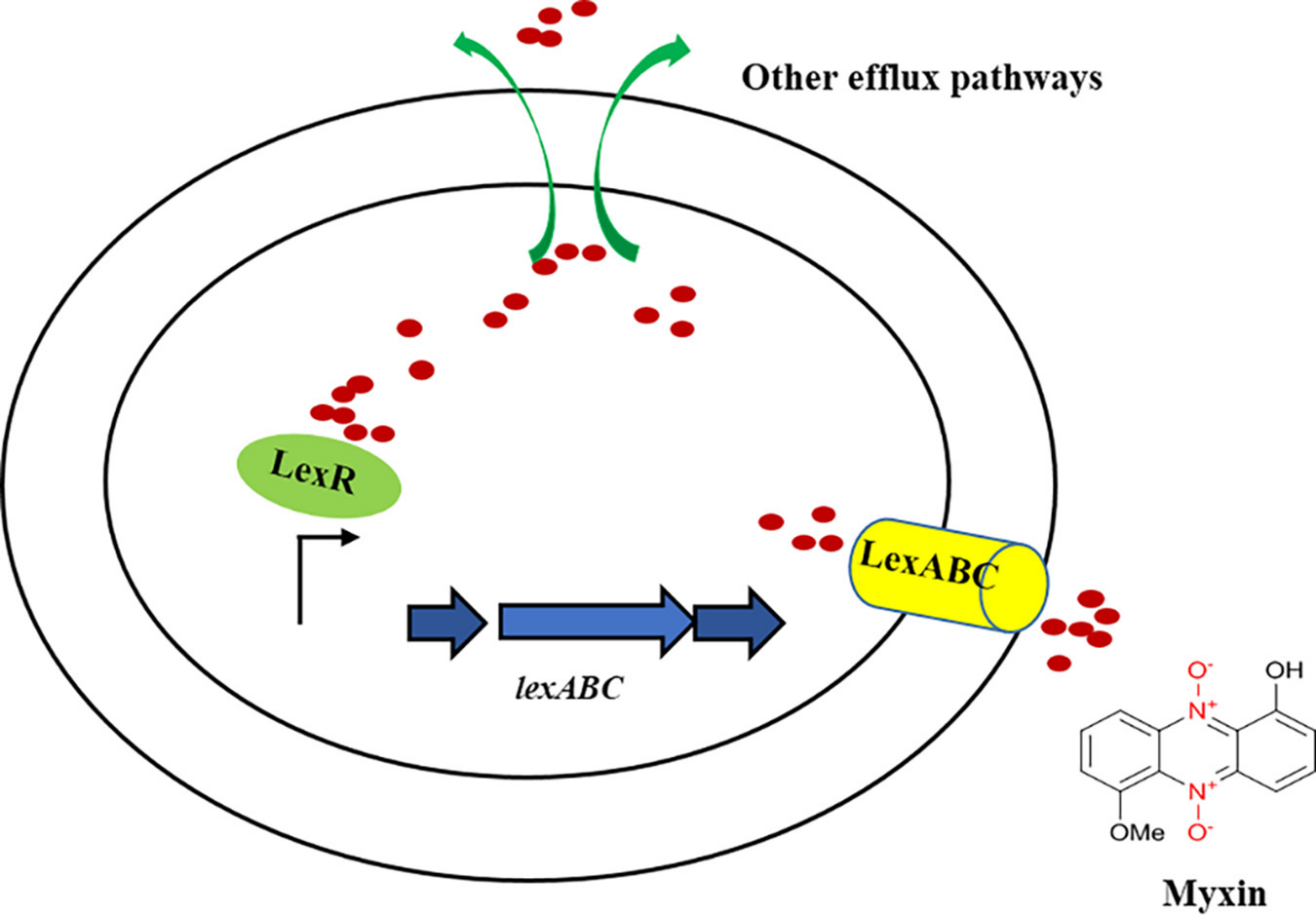
Proposed model for LexR regulation of the LexABC pump in myxin efflux of *L. antibioticus*. LexR senses and binds to myxin and then directly upregulates the LexABC pump. This pump increases the transport of myxin, affording self-protection to *L. antibioticus*. The red circle represents myxin.

Overall, we have defined a regulatory mechanism for myxin efflux in *L. antibioticus*. The LysR family regulator LexR binds with myxin and directly upregulates the LexABC pump (Fig. 8). This pump increases the transport of myxin, affording self-protection to *L. antibioticus*.

## MATERIALS AND METHODS

### Validation of the *lexQSABC* operon by RT-PCR.

*L. antibioticus* strain OH13 was grown at 28°C overnight in nutrient broth (3 g beef extract, 1 g yeast extract, 5 g tryptone, 10 g sucrose [pH = 7.0 to 7.2] in 1 liter distilled water) as seed culture. Then, a 1% (vol/vol) seed culture was transferred to a 1/10 dilution of tryptic soy broth (TSB). *L. antibioticus* OH13 cells were immediately harvested at an optical density at 600 nm (OD_600_) of 1.0 via centrifugation at 4,000 rpm for 10 min. Total RNA was extracted using a bacterial RNA extraction kit (Omega Bio-Tek, Norcross, GA) and quantified using an Eppendorf BioPhotometer Plus. Subsequently, Vazyme HiScript II Q RT SuperMix was used to eliminate gDNA and produce cDNA from 250 ng of RNA. Specific primers for each fragment are provided in Table S1 in the supplemental material. Standard PCR used TransTaq-T DNA polymerase with cDNA as the template, RNA and H_2_O as the negative control, and gDNA as the positive control.

### Purification of LexR and mutants.

The gene *lexR* was amplified from gDNA of OH13 with primers *lexR*-PF and *lexR*-PR (Table S1). The PCR product was digested by BamHI and EcoRI and cloned into plasmid pET-28a to obtain a pET-*lexR* construct. The *lexR* sequence was confirmed by DNA sequencing. The expression construct pET-*lexR* was introduced into E. coli BL21(DE3). The BL21-pET-*lexR* overnight culture was transferred to 100 mL LB medium containing kanamycin (25 μg/mL) and grown until an OD_600_ of 0.6. Isopropyl β-d-1-thiogalactopyranoside was added to the culture to a final concentration of 0.5 mM, and cells were cultured at 37°C for an additional 6 h. Cells were harvested and resuspended in 20 mL buffer (50 mM Tris-Cl, 300 mM NaCl; pH 7.9) and then lysed ultrasonically on ice. The supernatant was then loaded onto a Ni-nitrilotriacetic acid column previously calibrated with 5 mM imidazole buffer. His_6_-tagged LexR protein (53.1 kDa) was purified using an imidazole step gradient and detected with SDS-PAGE. Protein with His_6_ tag was concentrated and measured with Bradford dye reagent (Bio-Rad) and then used in EMSAs. Residue mutants LexR-V146A, LexR-K195A, and LexR-V146A-K195A were obtained using the appropriate mutant genes in the purification scheme. The site mutation for LexR was constructed through direct gene synthesis.

### EMSA.

The promoter region of the *lexQSABC* operon was predicted with the online tool BPROM (http://www.softberry.com/berry.phtml?topic=bprom&group=programs&subgroup=gfindb). A 165-bp DNA fragment containing the *lexQSABC* promoter region and a 158-bp promoter region from another RND efflux pump operon as control were amplified via PCR with 5′-end biotin-labeled primers. We also synthesized four pairs of complementary oligonucleotide 5′-end biotin-labeled primers. Primers were mixed with 1× annealing buffer (10× annealing buffer; 100 mM Tris-HCl, 1 M NaCl, 0.1 mM EDTA [pH 8.0]) and annealed at 95°C for 5 min. The products were kept at room temperature for 2 h, and subsequently we obtained the probes. Probe 1 was a 58-bp sequence from the *lexQSABC* promoter, and probe 2 was a 50-bp sequence without the putative binding site of LexR. Probe 3 was a 58-bp sequence from the 158-bp control probe, and probe 4 was a 58-bp sequence replacing the putative binding site of LexR to probe 3. The sequences of probes are shown in the supplemental material. EMSA was conducted using LightShift chemiluminescent EMSA kits (Thermo Scientific). The manufacturer’s instructions were followed for the EMSA, with binding reaction mixtures (20 μL) containing 1× binding buffer, 50 ng/μL poly(dI-dC), 2.5% (vol/vol) glycerol, 5 mM MgCl, 0.05% (vol/vol) NP-40, 5 fmol labeled probe, and different concentrations of LexR and myxin as required. Reactions were continued for 30 min at room temperature, and subsequent steps were completed following the manufacturer’s instructions. Chemiluminescence signals of biotin-labeled probes were captured using a Tanon 4600 imaging system (China). The primers are listed in Table S1.

### SPR analysis.

The binding affinity of myxin for LexR and its mutants was investigated via SPR using a bScreen LB 991 label-free microarray system (Berthold Technologies, Germany). Myxin was immobilized on photo cross-linker SPRi sensor chips, and LexR and mutant proteins were diluted separately with running buffer phosphate-buffered saline with 0.1% Tween 20 (PBST; pH 7.4) at concentrations of 10, 40, 160, 640, and 2,560 nM. The injection time was >600 s at a flow rate of 0.5 μL/s for each successive stage. Chips were then washed with running buffer for 360 s at a flow rate of 0.5 μL/s in each dissociation stage. Chip surfaces were regenerated to remove any remaining bound material with a pulse of 10 mM glycine-HCl (pH 2.5) at 20 μL/min for 300 s at the end of each association-dissociation cycle.

### Complementation and site-directed mutation of LexR.

For complementation of *lexR*, the target gene was amplified with primers *lexR*-CF and *lexR*-CR and then cloned into the plasmid pBBR1-MCS5. To construct the LexR site-directed mutants V146A or K195A, two sequences were obtained with primers (pBBR-lexR-F/146-1-R and 146-2-F/pBBR-lexR-R for V146A, pBBR-lexR-F/195-1-R and 195-2-F/pBBR-lexR-R for K195A), and then assembled with the plasmid pBBR1-MCS5. The expression constructs were confirmed by PCR and DNA sequencing and subsequently introduced into *lexR* deletion mutants by electroporation. The resultant strains were validated by PCR. The primers are listed in Table S1.

### qRT-PCR.

Bacterial cells were cultured in 1/10 TSB and collected at an OD_600_ of ~1.0. RNA extraction was carried out with a bacterial RNA extraction kit (Yeasen MolPure, China). The concentration and quality of RNA were detected with an Eppendorf BioPhotometer Plus. cDNA synthesis was performed with 250 ng of RNA using a kit (Vazyme HiScript II Q RT SuperMix with gDNA wiper). Primers for qRT-PCR designed with primer 3 online are shown in Table S1, and the 16S rRNA gene was used as a reference. The qRT-PCRs was performed on a Quantstudio 6 Flex system (Applied Biosystems) using ChamQ SYBR qPCR master mix (Vazyme). The experiment was repeated three times, each time in triplicate. Relative expression was analyzed using the threshold cycle (2^−ΔΔ^*^CT^*) method.
