# Supplementary material for: LexR Positively Regulates the LexABC Efflux Pump Involved in Self-Resistance to the Antimicrobial Di-N-Oxide Phenazine in Lysobacter antibioticus
Source: Microbiol Spectr. 2023 May 11;11(3):e04872-22. doi: 10.1128/spectrum.04872-22 (PMC10269722; doi:10.1128/spectrum.04872-22)
Supplement: Supplemental file 1 — Supplemental material. Download spectrum.04872-22-s0001.docx, DOCX file, 0.5 MB [file spectrum.04872-22-s0001.docx]

| **TABLE S1** Primers used in this study |
| --- |
| Name Primer sequence (5′→3′) Use or length |
| **Primers for RT-PCR** |
| Fragment 1-F AGCTGGCCTTGAGCACTTTC 204 bp  Fragment 1-R CATTCCGGGAATGACACTCG  Fragment 2-F CGGAACGGGCGAAGTAGTTG 168 bp  Fragment 2-R AAATCGGCTTCAACCGATGC  Fragment 3-F CGGCGAAGATCACGATCGAC 408 bp  Fragment 3-R CGTGCAGAAGGTGTTCTTCC  Fragment 4-F TCTGCCAGAACTCGGCATTG 280 bp  Fragment 4-R TGTTCGGCCTGTTCCTGACC |
| **Primers for probes amplification** |
| P*lex-*F ATGGGAGAAACGCGAATCGG 165 bp  P*lex-*R GGGCATTCAAGGAGGCAGAG |
| P*control*-F ATGCGCCTCGCGATCAACAC 158 bp  P*control*-R CGTAGGCGCACTACGCAATC |
| Probe1-F ACTTGATTAGCCACTGATTCAGGGAATAA 58 bp  TTGTCCCGACATCGGGCCAATGCCCTCTC  Probe1-R GAGAGGGCATTGGCCCGATGTCGGGACAA  TTATTCCCTGAATCAGTGGCTAATCAAGT |
| Probe2-F ACTTGATTAGCCACTGATTCAGGGACCC 50 bp  GACATCGGGCCAATGCCCTCTC  Probe2-R GAGAGGGCATTGGCCCGATGTC  GGGTCCCTGAATCAGTGGCTAATCAAGT |
| Probe3-F GGCTCCGCGCCTGCCAGCTTCTTTTTTCG 58 bp  TTGTCTGGGGGCTAGGGCGAGACACCCGC  Probe3-R GCGGGTGTCTCGCCCTAGCCCCCAGACAA  CGAAAAAAGAAGCTGGCAGGCGCGGAGCC |
| Probe4-F GGCTCCGCGCCTGCCAGCATAATTGTTCGT 58 bp  TGTCTGGGGGCTAGGGCGAGACACCCGC  Probe4-R GCGGGTGTCTCGCCCTAGCCCCCAGACA  ACGAACAATTATGCTGGCAGGCGCGGAGCC |
| **Primers for LexR complementation and site-directed mutation** |
| lexR-CF CGGGATCCAGCGCGATGATGAAGGCCAG *lexR* complementation  lexR-CR GCTCTAGAAGCTACGATGCTGCCTATCC |
| pBBR-lexR-F cccgggctgcaggaaATGGGAGAAACGCGAATCGG  146-1-R gggcaggtcgcctgcCCGCAGCGCCACGTC mutation of *lexR* at 146 residue  146-2-F gacgtggcgctgcggGCAGGCGACCTGCCC  pBBR-lexR-R ccgccaccgcggtggTCACGCCAGCGCCGC |
| pBBR-lexR-F cccgggctgcaggaaATGGGAGAAACGCGAATCGG  195-1-R gttgcggcgcgaggcCGGCATCGCCAGGGTG mutation of *lexR* at 195 residue  195-2-F accctggcgatgccgGCCTCGCGCCGCAACAA  pBBR-lexR-R ccgccaccgcggtggTCACGCCAGCGCCGC |
| **Primers for protein expression** |
| *lexR*-PF CGCGGATCCCCATGGCTCACGATCTCAAC 1155 bp  *lexR*-PR CCGGAATTCTCACGCCAGCGCCGCGTCCT |
| **Primers for qRT-PCR** |
| 16S-qF ACGGTCGCAAGACTGAAACT target 16S rDNA gene  16s-qR AAGGCACCAATCCATCTCTG  *lexA*-qF AGCAACACGCTCTGCTGATA target lexA gene  *lexA*-qR GACTGGTCGTTCCTCGACAT |

***

***

**FIG S1** LexR shows specific binding to *lexQSABC* promoter region. Lanes 1 and 2, the 165-bp *lex* labeled probe. Lanes 3-7, 158-bp labeled probes from a different RND efflux pump operon with different concentrations of LexR as a control. LexR couldn’t bind to the 158 bp probe. The labeled probes were added 5 fmol.


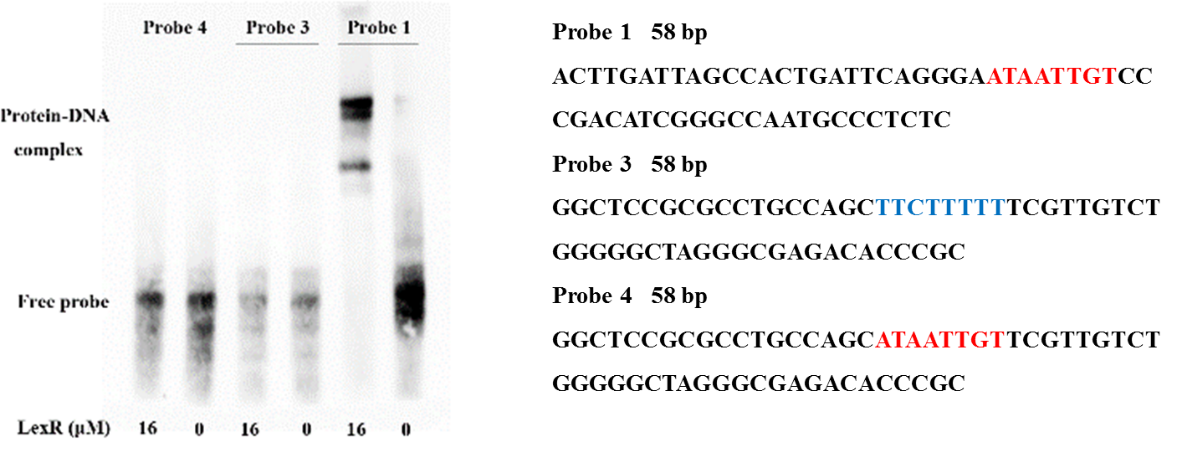


**FIG S2** The putative binding sequence is necessary for LexR. Probe 1, a 58-bp Biotin-labeled probe from *lexQSABC* promoter region; Probe 3, 58 bp from the unrelated promoter region in Fig. S1; Probe 4, replace the blue sequence of probe 3 with the eight base pair of putative binding sequence. The sequence of probes were presented in supplementary materials.

***Lex* probe sequence 165 bp**

ATGGGAGAAACGCGAATCGGGCTTGTCGGGGGCTCAACATGGCGGGGGGCCTCGTGTCGATCTGCGGCAAAGAGTAGAGAGATGGACGGCACTTGATTAGCCACTGATTCAGGGAATAATTGTCCCGACATCGGGCCAATGCCCTCTCTGCCTCCTTGAATGCCC

**Control probe sequence 158 bp**

ATGCGCCTCGCGATCAACACGGTCGCATCCCCTCTTTGCCAGCCACGGCTCCGCGCCTGCCAGCTTCTTTTTTCGTTGTCTGGGGGCTAGGGCGAGACACCCGCATACCGATGACTTCGTCGGCAGTGGCCCAAGACGGATTGCGTAGTGCGCCTACG

**Probe 1 sequence 58 bp**

ACTTGATTAGCCACTGATTCAGGGAATAATTGTCCCGACATCGGGCCAATGCCCTCTC

**Probe 2 sequence 50 bp**

ACTTGATTAGCCACTGATTCAGGGACCCGACATCGGGCCAATGCCCTCTC

**Probe 3 sequence 58 bp**

GGCTCCGCGCCTGCCAGCTTCTTTTTTCGTTGTCTGGGGGCTAGGGCGAGACACCCGC

**Probe 4 sequence 58 bp**

GGCTCCGCGCCTGCCAGCATAATTGTTCGTTGTCTGGGGGCTAGGGCGAGACACCCGC
